# Effects of Pharmacological Dose of Vitamin C on MDA-MB-231 Cells

**DOI:** 10.3390/biomedicines13030640

**Published:** 2025-03-05

**Authors:** Lunawati Lo Bennett

**Affiliations:** College of Pharmacy, Union University, Jackson, TN 38305, USA; llbennett@uu.edu; Tel.: +1-731-661-5921

**Keywords:** triple-negative breast cancer, vitamin C, caspases, MDA-MB-231, pharmacological dose, MCF7 cells, HEK293 cells, CCL205 cells

## Abstract

**Background/Objectives:** In 2022, approximately 2.3 million women were diagnosed with breast cancer worldwide, resulting in 670,000 deaths, which accounted for 6.9% of all cancer-related deaths. In the United States, 1 in 8 women will be diagnosed with breast cancer during their lifetime. It was estimated that 2024 would identify about 310,720 women and 2800 men diagnosed with invasive breast cancer. The future global burden of breast cancer is projected to rise to over 3 million new cases and 1 million deaths by 2040. Approximately 20% of breast cancer diagnoses are triple-negative breast cancer (TNBC), a type of cancer that lacks receptors for estrogen (ER-negative), progesterone (PR-negative), and human epidermal growth factor receptor 2 (HER2/neu-negative). Consequently, TNBC does not respond to hormonal or targeted therapies, making it challenging to treat due to its rapid growth, metastasis, and high recurrence rate within the first three years of therapy. Alternative chemotherapies are needed to address this problem. A pharmacological dose of vitamin C (high-dose VC) has been identified as a potential treatment for some cancer cells. The present study aimed to evaluate whether VC has a therapeutic effect on TNBC, using MDA-MB-231 cells as the model. Additionally, VC’s effects were trialed on other cancer cells such as MCF7 and on non-cancerous kidney HEK 293 and lung CCL205 cells. **Methods:** The MTT assay, Hoechst 33342 staining, nuclear-ID red/green staining, Rhodamine 123 staining, and Western blot analysis were employed to test the hypothesis that a pharmacological dose of VC can kill TNBC cells. **Results:** The upregulation of Apaf-1 and caspases -7, -8, and -9, the inhibition of matrix metalloproteinases (MMP-2 and MMP-9), a reduction in cell cycle protein expression, and the enhancement of tumor suppressor proteins such as p53 and p21 indicate that a pharmacological dose of VC has promising anti-cancer properties in the treatment of breast cancers. **Conclusions:** Pharmacological dose of VC exerts significant anti-cancer effects in MDA-MB-231 cells by promoting apoptosis, inhibiting metastasis, disrupting cell cycle progression, and enhancing tumor suppressor activity.

## 1. Introduction

Breast cancer remains one of the most prevalent and concerning diseases affecting women worldwide. Approximately 2.3 million women have been diagnosed with breast cancer worldwide, with 670,000 deaths, accounting for 6.9% of all cancer-related deaths. In the United States, 1 in 8 women will be diagnosed with breast cancer during their lifetime. It was estimated that, in 2024, 310,720 women and 2800 men would be diagnosed with invasive breast cancer [1,2,3]. Due to its complex nature and significant impact on public health, extensive research efforts are dedicated to understanding, treating, and ultimately eradicating this disease. Breast cancer consists of various subtypes, one of which is triple-negative breast cancer (TNBC). TNBC lacks receptors for the hormone estrogen (ER-negative), progesterone (PR-negative), and human epidermal growth factor receptor 2 (HER2/neu-negative). Consequently, TNBC does not respond to hormonal or targeted therapies. In addition to TNBC, other invasive breast cancers that express estrogen or progesterone receptors are classified as basal-like or luminal-like cancers. Approximately 20% of breast cancer diagnoses are TNBC [1,2].

Several characteristics make TNBC particularly challenging to treat. Firstly, TNBC often grows rapidly and undergoes metastasis, the process by which cancer cells spread from their primary site to distant parts of the body. Metastasis is considered the most life-threatening aspect of cancer, responsible for approximately 90% of cancer-related deaths in humans [3]. Secondly, TNBC can easily become resistant through the activation of mutated pathways, making it difficult to treat. Consequently, oncologists often use high doses of anti-cancer agents from multiple drug classes to prevent and treat metastasis and resistance. Thirdly, TNBC has a high recurrence rate within the first three years of therapy, with a five-year survival rate of 65% as compared to 86% for all breast cancers [2,3].

Because TNBC does not respond to hormonal or targeted therapies, treatment options are limited to traditional anti-cancer drugs, which often cause unfavorable adverse effects such as nausea, vomiting, bone marrow toxicity, cardiac arrest, renal failure, and infertility [4]. Therefore, several attempts have been made to find different compounds to alleviate this problem.

Vitamin C (ascorbic acid or ascorbate, VC) has been known for treating and preventing scurvy and the common cold since 1928 [5]. In the 1970s, several studies suggested the benefit of high-dose VC in improving advanced cancer survival [6]. However, a controversy over clinical results between Linus Pauling, a Nobel Prize recipient in chemistry, and the Mayo Clinic at that time led to the dismissal of VC as a promising anti-cancer agent. In recent times, this new knowledge has re-emerged, prompting investigations into the mechanism of action of VC as a possible anti-cancer agent [7,8,9,10].

A pharmacological dose of VC refers to a high dose VC that is significantly greater than the recommended daily allowance (RDA), which is typically consumed through diet and standard supplementation. These doses are often administered intravenously (IV) rather than orally to achieve higher plasma concentrations that are thought to have therapeutic effects in cancer treatment. The oral intake of VC is limited by intestinal absorption, which can only achieve plasma concentrations of up to about 250 micromolar. In contrast, IV administration can achieve plasma concentrations in the millimolar range. This is often used in the range from 10 g to 100 g per infusion. At high concentrations, VC can act as a pro-oxidant rather than an antioxidant. This effect generates hydrogen peroxide and other reactive oxygen species (ROS) that can damage cancer cells more than normal cells. Pharmacological doses of VC are generally well tolerated, with potential side effects including gastrointestinal discomfort, kidney stones (particularly in individuals with a history of kidney stones), or rare complications related to IV administration [11].

The MDA-MB-231 cell line, derived from a pleural effusion of a patient with invasive ductal carcinoma, is frequently utilized to investigate advanced stages of breast cancer. The cells are characterized as ER-negative and PR-negative. Microarray profiling indicates that the MDA-MB-231 cell genome aligns with the basal subtype of breast cancer. It also lacks HER2/neu, making it an effective model for TNBC. These cells are commonly used to study metastasis, tumor progression, and drug resistance mechanisms in breast cancer [12,13].

The aim of this study was to determine the pharmacological dose of vitamin C (VC) required to induce cell death in MDA-MB-231 cells and to elucidate the molecular pathways involved in the process in triple-negative breast cancer (TNBC) cells. Additionally, the effects of VC were evaluated in MCF cells, which are estrogen and progesterone receptor-positive, HER2/neu receptor-negative, and considered a less invasive form of breast cancer. This study also examined the impact of VC on non-cancerous lung fibroblasts CCL205 cells, kidney HEK293 cells, and rat spleen to assess the specificity of its cytotoxic effects on cancerous versus non-cancerous cells.

## 2. Materials and Methods

To provide a concise overview of the research design, procedures, and results, a simplified scheme is presented below.

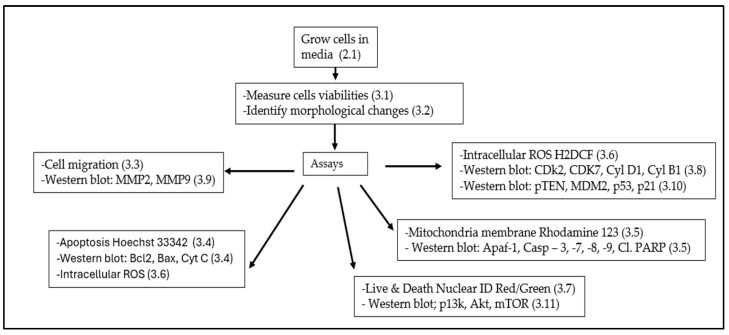



### 2.1. Cell Culture, Media, and Rat Tissue

MDA-MB-231 human breast adenocarcinoma cell lines (ATCC, HTB-26) were cultured to investigate the cytotoxic effects of vitamin C on triple-negative breast cancer (ATCC, Manassas, VA, USA). These cells were grown in Dulbecco’s Modified Eagle’s Medium (DMEM). This contained 4 mM L-glutamine, 4500 mg/L glucose, 1 mM sodium pyruvate, and 1500 mg/L sodium bicarbonate (ATCC), and was supplemented with 10% fetal bovine serum (FBS, ATCC) and 1% penicillin/streptomycin (100 U/mL penicillin, 200 µg/mL streptomycin) (Sigma Aldrich, St. Louis, MO, USA). The cells were maintained at 37 °C in a humidified atmosphere of 5% CO_2_.

To compare vitamin C’s effects on different breast cancer subtypes, MCF-7 cells (ATCC, HTB 22), which represent estrogen and progesterone receptor-positive, HER2/neu-negative breast cancer, were cultured in Eagle’s Minimum Essential Medium (EMEM, ATCC) under similar conditions, with identical FBS and penicillin/streptomycin supplementation.

For non-cancerous controls, human epithelial kidney (HEK 293, ATCC CRL-11268) and lung fibroblast (ATCC, CCL 205) cell lines were cultured in DMEM and EMEM, respectively, in the same conditions. Most of the cell passages ranged between 5 and 12. Additionally, rat spleen cells, provided by Dr. W. Thierfelder from Union University’s Biology Department, were prepared for cell viability assays to further evaluate the selectivity of VC’s cytotoxic effects.

### 2.2. Cell Viability Assay

Cell viability was assessed using the MTT (3-[4,5-dimethylthiazol-2-yl]-2,5-diphenyl-tetrazolium bromide, Sigma Aldrich) method, as previously described, to determine the concentration of VC required to kill about 20% of cells or 50% of cells (IC50) [13]. Cells were detached with trypsin/EDTA (Sigma Aldrich) and diluted to a concentration of 1 × 10^5^ cells/mL in culture medium, and 100 µL of the cell suspension was plated into each well of a 96-well plate (Greiner-Bio One, Monroe, NC, USA). Following overnight incubation at 37 °C in 5% CO_2_, the medium was replaced with neutralized VC at concentrations ranging from 0.25 mM to 125 mM and further incubated for 24 h at 37 °C. Subsequently, the medium was aspirated, wells were rinsed with 100 µL of DPBS, and 50 µL of MTT solution was added for a 4 h incubation. Cells were then lysed with 150 µL of dimethyl sulfoxide (DMSO, Amresco, Solon, OH, USA), and absorbance was measured at 570 nm using a spectrophotometer (Molecular Devices LLC, San Jose, CA, USA). In addition to MDA-MB-231 cells, the assay was also conducted on MCF7 and rat spleen cells.

### 2.3. Morphology Analysis

The morphological changes in MDA-MB-231 cells treated with VC for 0 and 24 h were documented using an inverted microscope with 40× capacity (Motic AE31, Hongkong), following established protocols [14,15]. Besides MDA-MB-231, the assay also was performed on MCF7, HEK 293, and CCL 205 cell lines.

### 2.4. Cell Migration Assay

To evaluate if VC affects cellular migration, MDA-MB-231 cells were cultured at a density of 1 × 10^5^ cells in a 12-well dish for 24 h. Subsequently, a scratch was created using a sterile pipette tip, and the area of migration was captured using an inverted microscope (40×). The width of the scratch was quantitatively analyzed at 0 and 24 h post-scratch, following established methodologies [14,15]. Besides MDA-MB-231, the assay was also performed on MCF7, HEK 293, and CCL 205 cell lines.

### 2.5. Apoptosis Assay

To examine nuclear chromatin morphological alterations in MDA-MB-231 cells after VC treatment, the NucBlue™ Live Cell Hoechst 33342 (Life Technologies, Carlsbad, CA, USA) staining assay was conducted according to established protocols [14,15]. The quantity of apoptotic and non-apoptotic cells was recorded using the Floid Cell Imaging Station with a scale bar of 100 µm (Life Technologies, Carlsbad, CA, USA), and fluorescence intensities were assessed using Image J software Fiji Linus 64 (NIH, Bethesda, MD, USA). A histogram was constructed to compare the percentage changes in apoptotic cells across the treatment groups. Besides MDA-MB-231, assay was also performed on MCF7, HEK 293, and CCL 205 cells.

### 2.6. Mitochondrial Membrane Potential (ψ_m_) Assay

To identify if there were changed in mitochondrial membrane potential, cells were stained with Rhodamine 123 fluorescence probe (Invitrogen, Eugene, OR, USA) as previously described [14,15]. The relative intensities of green fluorescence were captured using FLoid cell imaging with a scale bar of 100 µm. A histogram was prepared to compare the quantities of fluorescence using Image J software. Besides MDA-MB-231, assay was also performed on MCF7, HEK 293, and CCL 205 cells.

### 2.7. Intracellular ROS Assay

To examine if intracellular reactive oxygen species (ROS) were generated in MDA-MB-231 cells after VC treatment, H_2_DCF-DA (2’7’-Dichloro-dihydrofluorescein diacetate, Invitrogen) staining assay was performed following the manufacturer protocol as previously reported [14,15]. The relative intensities were captured using Floid cell imaging station with a scale bar of 100 µm. A histogram was prepared to compare the quantities fluorescence using Image J software. Besides MDA-MB-231, assay was also performed on MCF7, HEK 293, and CCL 205 cells.

### 2.8. Live and Death Cells Assay

To assess whether the deoxynucleic acid (DNA) of MDA-MB-231 cells was altered following VC treatment, a nuclear-ID red/green cell viability assay was performed according to the manufacturer’s protocol (Enzo Life Sciences Inc, Farmingdale, NY, USA), as previously described [14]. The simultaneous use of red and green dyes allows for the determination of live and dead cells, which were captured using Floid cell imaging with a scale bar of 100 µm. A histogram was prepared to compare the quantities of live and dead cells using Image J software. Besides MDA-MB-231, assay was also performed on MCF7, HEK 293, and CCL 205 cells.

### 2.9. Western Blot Analysis

Western blot analysis was performed to determine changes in the level of proteins of interest after treatment of MDA-MB-231 cells with VC, as previously described [14,15]. Briefly, RIPA buffer supplemented with protease and phosphatase inhibitors (Sigma Aldrich) was used to extract proteins from cells followed by measurement of proteins concentrations with Bradford protein assay per manufacture protocol (Bio-Rad Laboratories, Hercules, CA, USA). Equivalent amounts of proteins 50 μg was loaded onto 10% polyacrylamide gels followed by electrophoresis. The gel was then transferred unto Immuno- Blot PVDF membranes using Trans Blot Turbo (Bio-Rad) for 30 min. The membranes were then blocked for 2 h with 5% dry milk dissolved in Tris-buffered saline containing 0.1% Tween-20 (TBST) at room temperature. Finally, the membranes were incubated overnight with specific primary antibodies from Cell Signaling Technology (Beverly, MA, USA) or from Santa Cruz Biotechnology Inc. (Santa Cruz, CA, USA). Caspase-3, -7, -8, -9, Cl. PARP, MMP -2, -9, mTOR, pTEN, p13k, p53, Akt, p21, Cyclin B1, Cyclin D1, Cyt C, CDK2 were from Cell Signaling Technology. β-actin, Bax, BCl2, Apaf-1, CDK7 were from Santa Cruz Technology. The following day, the membranes were then washed several times in TBST, followed by incubation for 2 h with secondary antibodies. The protein bands were developed using Enhanced Chemi Luminescence (ECL, Bio-Rad) Western Blotting detection reagents and the band pictures were taken using Bio-Rad ChemiDoc XRS^+^ Imaging system. Besides MDA-MB-231, assay was also performed on MCF7. Data for MCF7 can be found in the Supplemental file.

### 2.10. Statistical Analysis

All the statistical results were expressed as the mean ± SD of three independent sets of experiments. Differences between individual and combination treatment groups were analyzed using Newman–Keuls one-way ANOVA. ** *p* < 0.01 and *** *p* < 0.001 were considered statistically significant.

## 3. Results

### 3.1. Cell Viability Assay

**Figure 1:**
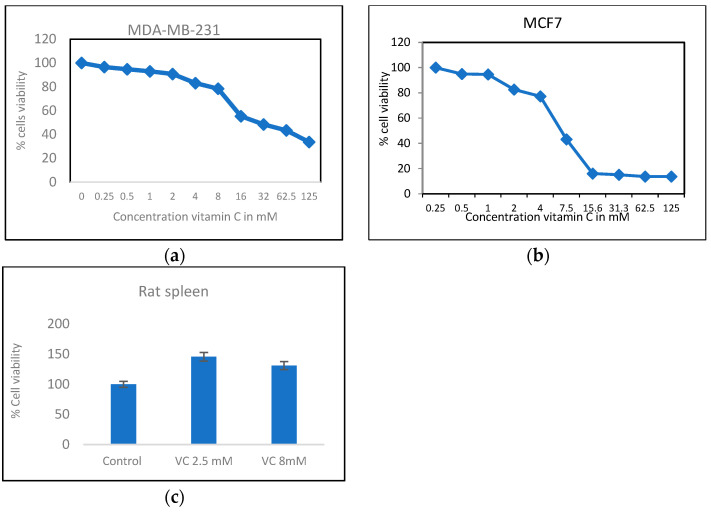
(**a**) MTT result of cell viability MDA-MB-231 cells treated with VC from 0 to 125 mM. (**b**) shows MCF7 cells treated with VC from 0 to 125 mM. (**c**) shows that rat spleen had higher % cell viability with treatment of VC at 2.5 mM and 8 mM showing non-cytotoxic effect of VC on normal cells.

The cell viability of MDA-MB-231 cells was assessed using the MTT assay with VC concentrations ranging from 0.25 mM to 125 mM. The concentration that resulted in approximately 20% and 50% cell death (IC50) was determined. VC at 8 mM reduced cells viability by 20%, while 16 mM caused 50% cell death in MDA-MB-231 cells. In MCF7 cells, the concentrations that killed 20% and 50% of the cells (IC50) were 4 mM and 7 mM, respectively. In contrast, an increase in viability was observed in rat spleen cells treated with 2.5 mM and 8 mM VC, indicating that VC did not cause cell death in normal cells such as spleen (Figure 1). 

### 3.2. Changed in Cell Morphologies

**Figure 2:**
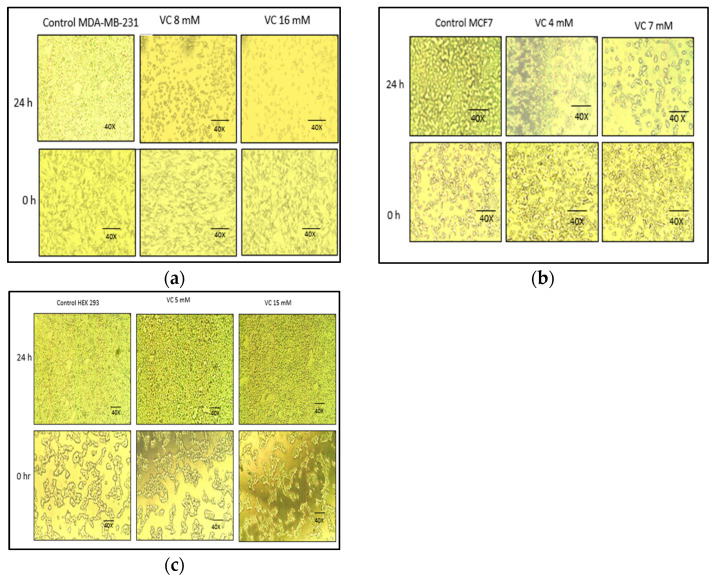
Inverted microscopic images of MDA-MB-231 and MCF7 cancer cells after 24 h of treatment with VC at concentrations of 8 mM and 16 mM for MDA-MB-231, and with VC at concentrations of 4 mM and 7 mM for MCF7. Higher concentrations of VC led to increased cell death in both MDA-MB-231 and MCF7 compared to their respective controls (**a,b**). In contrast, VC at 5 mM and 15 mM promoted cell growth in non-cancerous HEK-293 kidney cells (**c**). The scale bar represents 40×.

VC concentrations at 8 mM and 16 mM significantly altered the cell morphology compared to the control MDA-MB-231. As shown in Figure 2, control cells displayed fibroblast-like growth, adhering to the culture dish. In contrast, cells treated with 8 mM VC exhibited some cell death, while those treated with 16 mM showed increased cell death, with most cells floating in the media (Figure 2a). MCF7 control cells showed growth at 24 h, whereas treatment with 4 mM and 7 mM VC resulted in reduced growth and noticeable changes in cell morphology (Figure 2b).

Non-cancerous HEK 293 kidney cells exhibited increased growth when treated with 5 mM and 15 mM of VC, compared to the control, indicating that VC did not induce cell death in these non-cancerous cells.

### 3.3. Inhibition of Cell Migration

**Figure 3:**
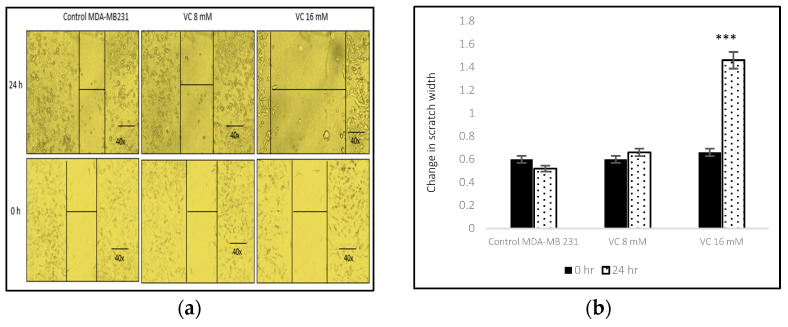

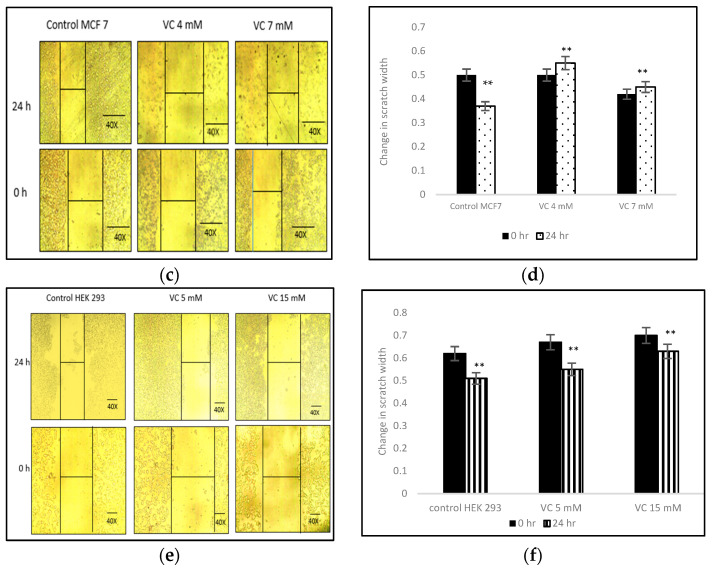
Image (**a**) displays inverted microscope images of cell migration following the 24 h treatment of MDA-MB-231 cells with VC 8 mM or 16 mM VC, while MCF7 cells received 4 mM and 7 mM of VC. In the control groups, a narrow scratch area indicated maximal cancer cell migration, whereas VC-treated cells exhibited a wider scratch area, reflecting reduced migration (**a**,**c**). Conversely, VC at 5 mM and 15 mM enhanced cell growth in non-cancerous HEK 293 kidney cells, as evidenced by a decreased scratch width (**e**). Figures (**b**,**d**,**f**) presents the corresponding histograms, with ** *p* < 0.01 and *** *p* < 0.001 (post hoc Newman–Keuls test) compared to the 0 h measurements of the corresponding control cells.

Following a 24 h treatment with 8 mM VC, significant changes in cell migration were observed in MDA-MB-231 cells, accompanied by a marked increase in cell death at 16 mM VC treatment as compared to the control. In the control group, a slight increase in cell migration suggested early signs of invasion and metastasis (Figure 3a,b). Similarly, MCF7 cells demonstrated a modest increase in cell death at 4 mM and 7 mM VC, though this effect was less pronounced than the significant cytotoxicity observed at 16 mM VC in MDA-MB-231 cells. Importantly, non-cancerous HEK 293 kidney cells treated with 5 mM or 15 mM VC for 24 h showed no significant changes in cell growth compared to the control, indicating that VC is non-cytotoxic to non-cancerous cells.

### 3.4. Induction of Apoptosis

**Figure 4:**
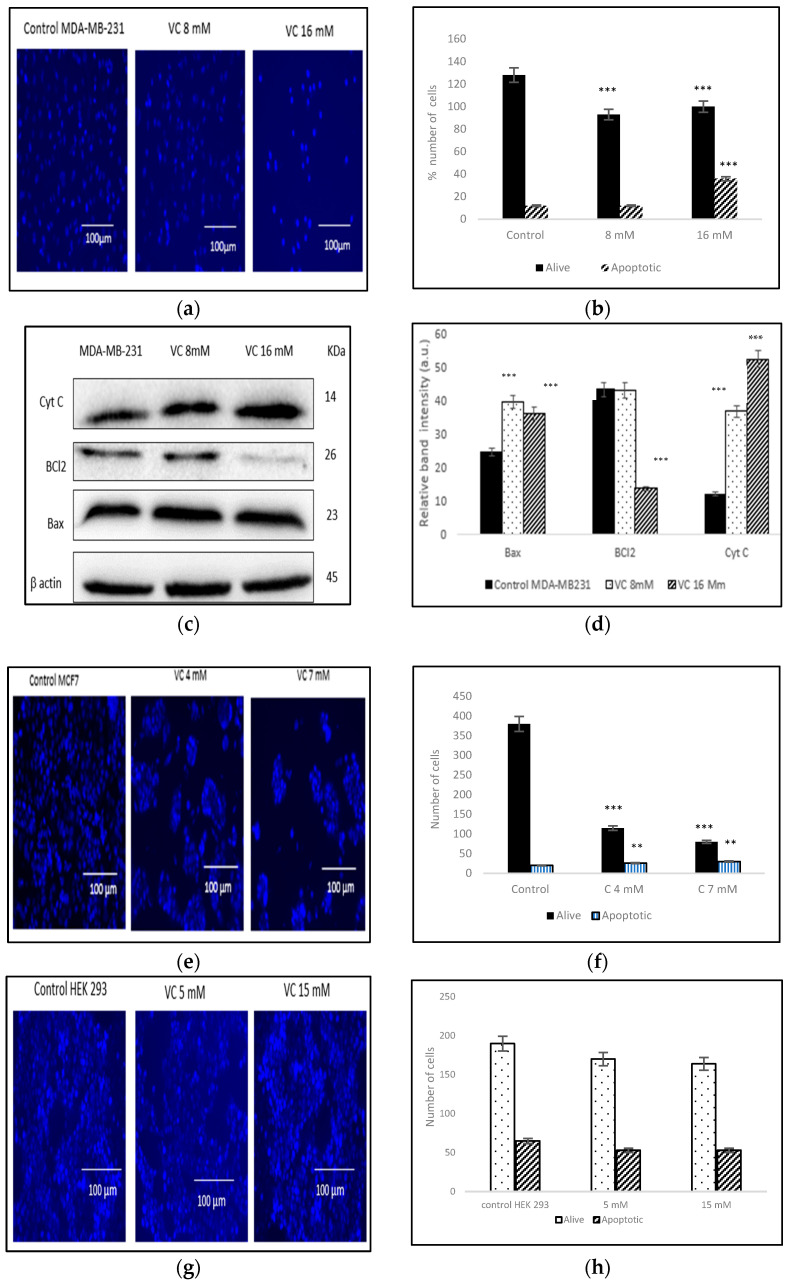

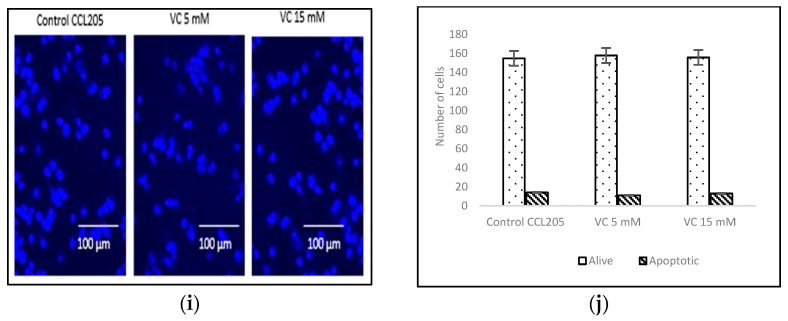
(**a**) displays fluorescence microscopy images of MDA-MB-231 control cells alongside cells treated with 8 mM and 16 mM VC for 24 h, followed by Hoechst 33342 staining. (**b**) illustrates a histogram comparing the percentage of apoptotic and live cells relative to the control. (**c**) presents Western blot analysis showing the expression levels of Bax, Bcl-2, and cytochrome C, while (**d**) provides a histogram comparing these expression levels to the control group. In MCF7 cells, fluorescence analysis revealed significant changes in the live-to-apoptotic cell ratio (**e**,**f**). In contrast, VC treatment at 5 mM and 15 mM in non-cancerous HEK 293 kidney cells or CCL 205 lung cells did not significantly affect this ratio, indicating no cytotoxic effect on non-cancerous cells (**g**–**j**). The scale bar represents 100 µm. Statistical significance was determined using the post hoc Newman–Keuls test ** *p* < 0.01, and *** *p* < 0.001) compared to the respective controls.

VC induced apoptosis in treated cells of MDA-MB-231, as observed using Hoechst 33342 staining. A higher concentration of VC at 16 mM resulted in increased apoptosis, as indicated by the bright blue fluorescence due to condensed and fragmented nuclei, whereas control cells exhibited lower fluorescence, signifying healthy cells (Figure 4a). The histogram in Figure 4b illustrates the difference in cell expression following VC treatment, as analyzed using ImageJ software. Additionally, Western blot analysis revealed changes in the expression of pro-apoptotic Bax and anti-apoptotic Bcl-2 proteins, suggesting intracellular ROS generation. Figure 4c showed a significant increase in Bax expression alongside a slight decrease in Bcl-2 expression with VC 8 mM and a more pronounced decrease with VC 16 mM. ROS generation also led to a reduction in mitochondrial membrane potential, causing cytochrome C release into the cytosol, which further triggered apoptosis. Figure 4d presents a histogram of the data from Figure 4c. Figure 4e-j represents effect of VC in cancer cells MCF7, non-cancerous HEK 293 cells, and non-cancerous CCL 205.

For Western blot analysis of MCF7, please refer to the Supplementary Material File.

### 3.5. Depletion of Mitochondrial Membrane Potential (ψ_m_)

**Figure 5:**
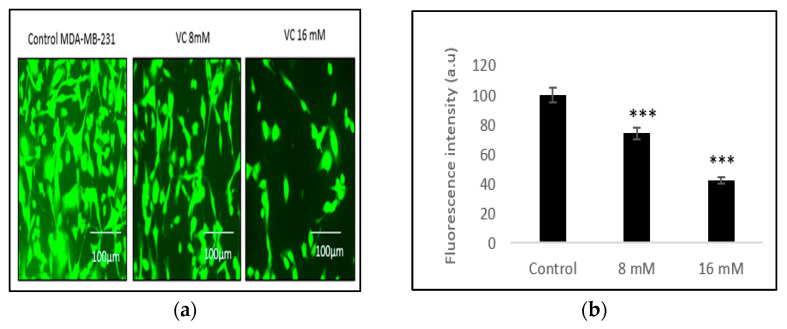

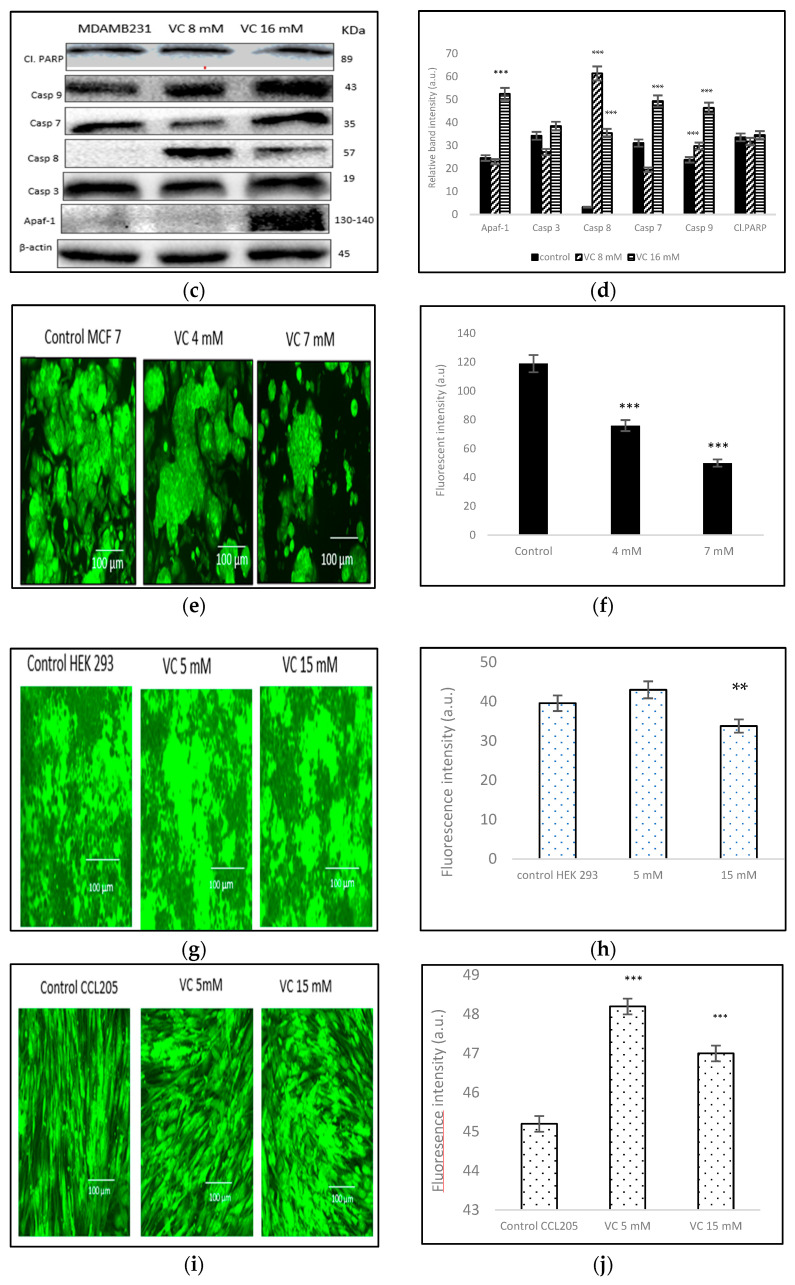
(**a**) Fluorescence microscopic images of MDA-MB-231cells after 24 h treatment with VC 8 mM or VC 16 mM and staining with Rhodamine 123. (**b**) Histogram represents percentage of cells changing in mitochondrial membrane potential, with higher VC showing pronounced decrease in membrane potential and darker cells compared to control. (**c**) Shows Western blot analysis of different signaling activity proteins involved in caspase cascade and Apaf-1. (**d**) Histogram represents upregulation of Apaf-1 and cas-3 and -9 in cells treated with VC 8 mM or 16 mM in MD-MBA-231 cells. Fluorescence analysis of MCF7 also showed pronounced decrease in cells treated with VC 4 mM and 7 mM, with the histogram representing the change in fluorescence (**e**,**f**). In contrast, VC treatment at 5 mM and 15 mM in non-cancerous HEK 293 and CCL 205 cells showed significant increase in membrane potential assuring favorable effect of VC in non-cancerous cells (**g**,**i**). The histograms show the effect of VC on HEK 293 and CCL 205 (**h**,**j**). Scale bar represents 100 µm. Significant change in mitochondrial membrane potential for treatment vs. control group, ** *p* < 0.01 and *** *p* < 0.001, post hoc Newman–Keuls test.

The depletion of mitochondrial membrane potential due to cell death was evident through the generation of reactive oxygen species (ROS) in MDA-MB-231 cells treated with VC, as detected by Rhodamine 123 staining. Control cells displayed higher fluorescence intensity, indicating a healthier membrane potential compared to treated cells. This depletion was most pronounced in cells treated with 16 mM VC (Figure 5a,b).

Western blot analysis further revealed an increased expression of apoptotic protease activating factor (Apaf-1), a cytoplasmic protein involved in the induction of apoptosis. Additionally, apoptosis within the mitochondria was marked by the activation of key proteins, including caspases -7, -8, and -9. The activation of these caspases represents a critical step in cancer cell apoptosis, with VC directly triggering both initiator caspases (caspases -8 and -9) and executioner caspase -7 (Figure 5c,d). MCF, HEK 293, CCL205 cells were also tested if they caused depletion in mitochondrial potential (Figure 5e,g,i). Corresponding histogram showed as Figure 5f,h,J.

### 3.6. Induction of Intracellular ROS

**Figure 6:**
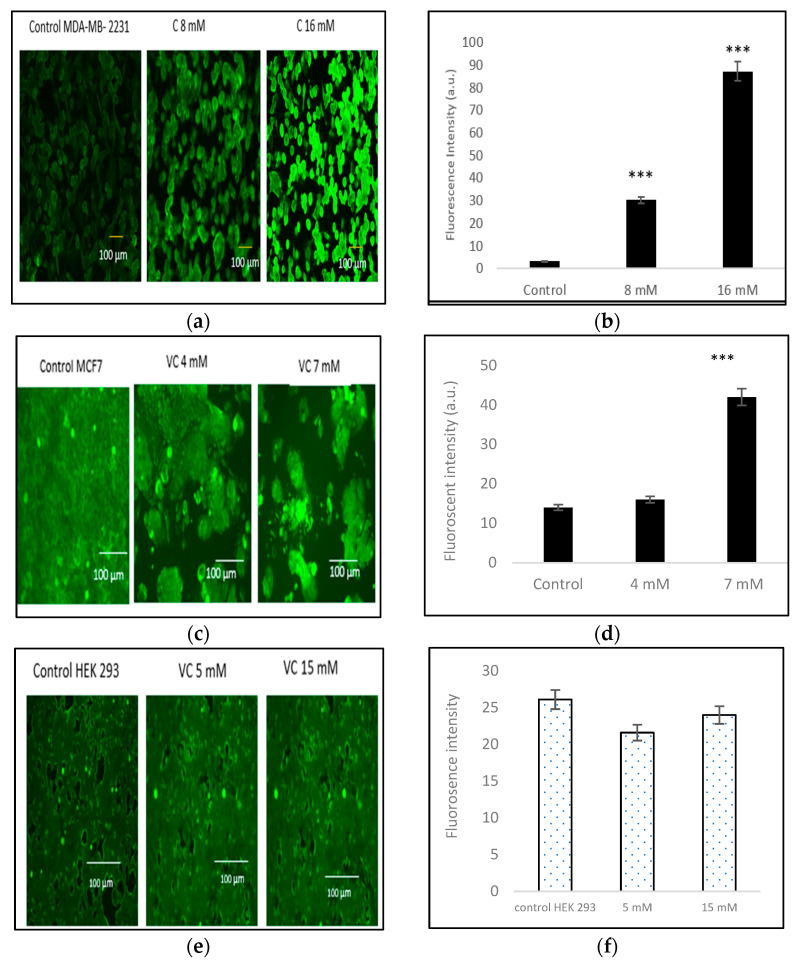

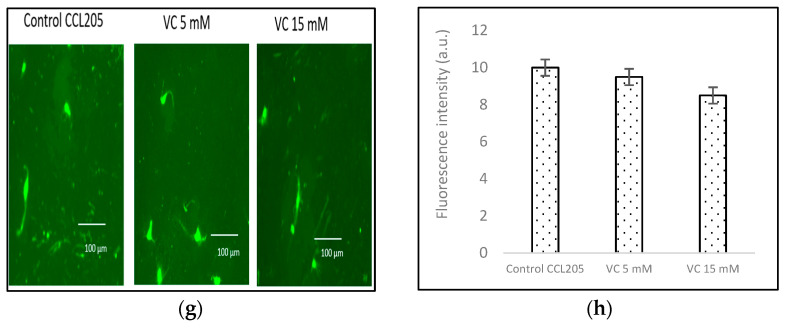
(**a**) shows the results of intracellular ROS generation in MDA-MB-231 cells using the H2DCFDA staining method. Control cells displayed minimal ROS formation, as indicated by darker green fluorescence. In contrast, cells treated with VC at 8 mM and 16 mM exhibited increased ROS levels, as characterized by progressively brighter green fluorescence. The intensity of ROS generation was directly proportional to the concentration of VC, with 16 mM showing the highest level of ROS. (**b**) presents a histogram comparing ROS levels in treated cells versus control. To further evaluate the effect of VC on different cell lines, ROS generation was also assessed in MCF7 cells and non-cancerous HEK 293 kidney and CCL 205 lung cells. MCF cells demonstrated a similar increase in ROS as MDA-MB-231 cells (**c**) with histogram presented (**d**). Non-cancerous HEK 293 and CCL 205 cells exhibited significantly lower levels of ROS in response to VC treatment, suggesting a minimal or non-cytotoxic effect of VC on non-cancerous cells (**e**–**h**). Scale bar indicated 100 µm. A significant change in intracellular ROS generation versus control, *** *p* < 0.001, post hoc Newman–Keuls test.

Intracellular ROS generation was assessed using the H2DCFDA staining method. Control MDA-MB-231 cells exhibited lower ROS levels, as indicated by a darker green fluorescence, compared to MDA-MB-231 cells treated with 8 mM or 16 mM VC. The higher the concentration of VC, the greater the intracellular ROS generation, characterized by a brighter green fluorescence (Figure 6a). Figure 6b presents a histogram comparing ROS levels in treated cells versus the control. To further evaluate the effects of VC on different cell lines, MCF cells, along with non-cancerous HEK 293 kidney cells and CCL 205 lung cells, were also tested (Figure 6c–h).

### 3.7. Induction of Cell Death

**Figure 7:**
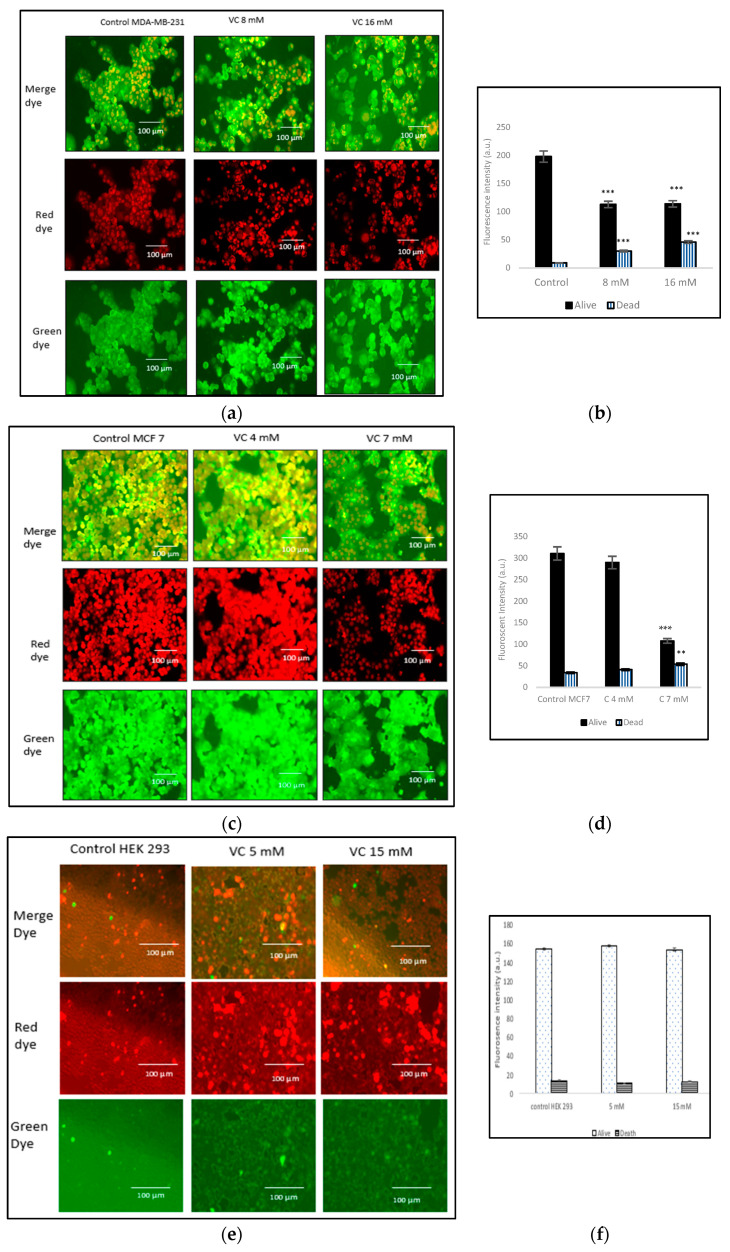

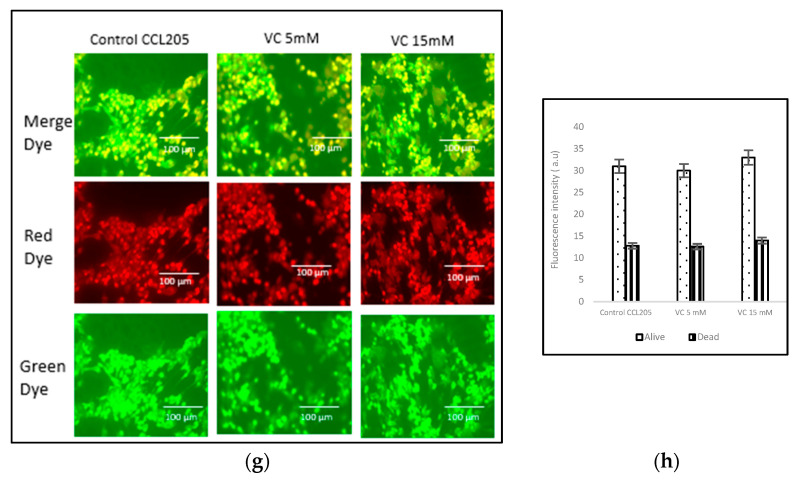
(**a**) Fluorescence microscopic images from alive and death nucleic acid staining using nuclear-ID red/green cell viability method. (**b**) The histogram represents the percentage of alive or death from cells treated with VC 8 mM or VC 18 16 mM vs. control of MDA-MB-231. (**c**–**h**) depict the effects of varying VC concentrations on cell viability in MCF7 cancer cells, non-cancerous HEK 293 kidney cells, and CCL 205 lung cells. MCF7 cells exhibited an increase in cell death at higher VC concentrations: 7 mM vs 4 mM (**c**,**d**). As the VC dose increased, a higher proportion of cells were stained red, reflecting the cytotoxic effects of VC. Control HEK 293 and CCL 205 cells versus cells treated with 5 mM or 15 mM VC displayed a similar proportion of live and dead cells, indicating that VC had no significant cytotoxic effect on these non-cancerous cell lines. The green fluorescence in both HEK 293 and CCL 205 cells was predominant, signifying high cell viability in both control and VC-treated groups (**e**–**h**). These findings highlight VC’s selective cytotoxicity, with higher concentrations inducing cell death in cancerous MDA-MB-231 and MCF7 cells, while non-cancerous HEK 293 and CCL 205 cells remained largely unaffected. Scale bar indicates 100 µm. Significant change in cells death due to VC treatment versus control, ** *p* < 0.01 and *** *p* < 0.001, post hoc Newman–Keuls test.

The viability of cells treated with two different concentrations of VC was assessed using the nuclear-ID red/green cell staining method. Live cells were stained green, as the dye penetrated the cytoplasm of intact cell membranes, while dying or dead cells, with compromised membranes, were stained red. In the control group of MDA-MB-231, cells displayed a red or orange color when the red and green dye images were merged, indicating non-viable cells. The treatment of MD-MBA-231 with 16 mM VC led to the highest number of cell deaths, as evidenced by more orange color observed after merging the red and green dyes (Figure 7a). Figure 7b presents a histogram showing cells dead versus live cells in MDA-MB-231, analyzed using ImageJ software. To further evaluate VC’s effects on various cell lines, MCF7 cells, along with non-cancerous HEK 293 kidney cells and CCL 205 lung cells, were also tested (Figure 7c–h).

### 3.8. Modulation of Cell Cycle Proteins

**Figure 8:**
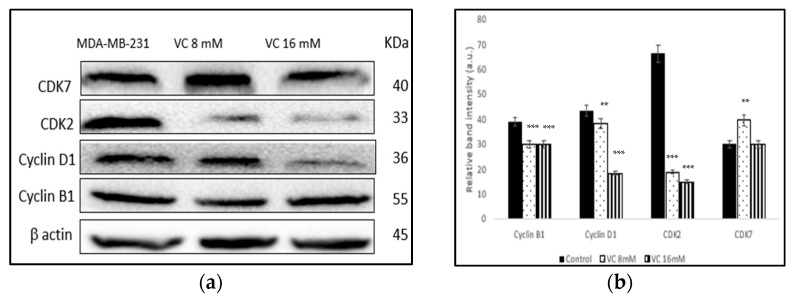
(**a**) Western blot analysis of proteins involved in cell cycle regulation. The expression of CDK2, cyclin D1, and cyclin B1 significantly decreased in cells treated with VC. (**b**) shows histogram downregulation of these proteins compared to its control, ** *p* < 0.01 and *** *p*< 0.001, post hoc Newman–Keuls test.

The inhibition of cell cycle regulatory proteins is considered an important strategy in the treatment of TNBC. Western blot analysis revealed a decrease in the band intensity of CDK2, cyclin B1, and cyclin D1 in MDA-MB-231, which play important roles in cell cycle regulation (Figure 8a). Figure 8b displays a comparison of the changes in cell cycle proteins, depicted as a histogram.

### 3.9. Inhibition of Matrix Metalloproteinases

**Figure 9:**
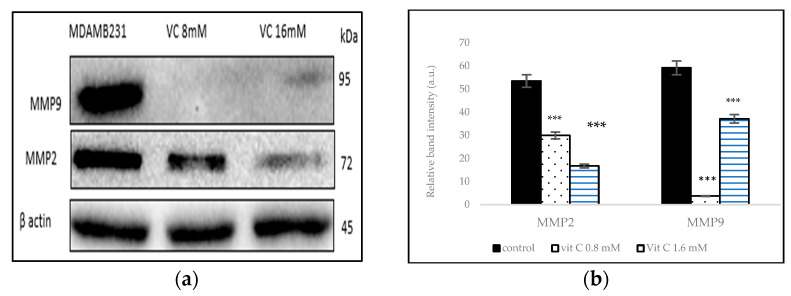
(**a**) Western blot analysis of MMP2 and MMP9 proteins. (**b**) Histogram represents downregulation of these proteins vs. control group. Significant decrease in protein expressions *** *p*< 0.001, post hoc Newman–Keuls test.

To determine if VC caused the inhibition of cell migration and invasion associated with matrix metalloproteinases (MMP)-2 and -9, proteins that are involved in angiogenesis, Western blot analysis was performed in MDA-MB-231 (Figure 9a). The band intensity histogram in Figure 9b shows the expression of MMP2 and MMP9 compared to the control.

### 3.10. Modulation of MDM2, -p53, and p21 Pathways

**Figure 10:**
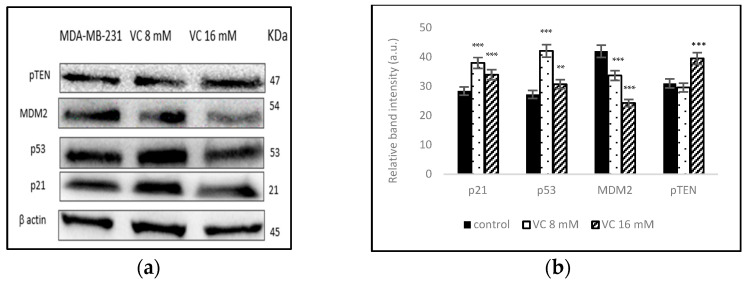
(**a**) Western blot analysis of p21, p53, pTEN, and MDM2 proteins. (**b**) Histogram represents the upregulation of p21, p53 and pTEN proteins and the downregulation of MDM2 as compared to the control. Significant change in protein expressions vs. control, ** *p* < 0.01 and *** *p*< 0.001, post hoc Newman–Keuls test.

To determine if VC regulated the cellular pathway involved in DNA repair, cell cycle, apoptosis, and angiogenesis, the interaction between p53 tumor suppressor protein and oncogene mouse double minute 2 homolog (MDM2) was studied using Western blot analysis. p21 gene codes for cyclin-dependent kinase inhibitor were upregulated along with p53 (Figure 10a). The band intensity histogram in Figure 10b shows the expressions of p53, p21, MDM2, and the phosphatase and tensin homologs deleted on chromosome ten (pTEN).

### 3.11. Inhibition of p13k/Akt/mTOR Pathway

**Figure 11:**
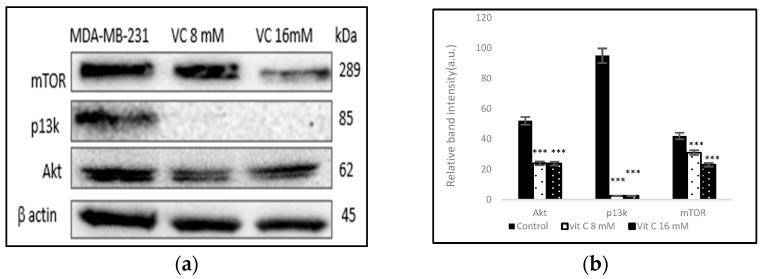
(**a**) Western blot analysis of p13k, Akt, and mTOR proteins. (**b**) Histogram represents the downregulation of p13k, Akt, and mTOR proteins from cells treated with VC as compared to the control. Significant change in protein expressions *** *p*< 0.001, post hoc Newman–Keuls test.

To determine if VC is involved in the regulation of the phosphoinositide 3 kinase (p13k)/Akt/mammalian target of rapamycin (mTOR), a complicated intracellular pathway that leads to cell growth and cancer proliferation, the interaction was studied using a Western blot assay. The band intensity histogram in Figure 11b shows the expressions of p13k, Akt, and mTOR compared to the control.

## 4. Discussion

**Figure 12:**
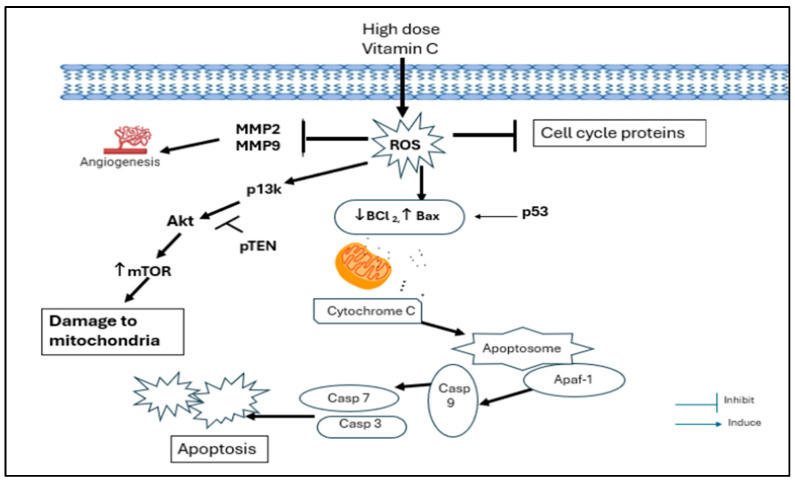
The proposed mechanism of action of a high dose of VC on MDA-MB 231 cells.

In the present study, a pharmacological dose of VC showed convincing evidence of killing MDA-MB-231, a TNBC model. At concentrations of 8 mM and 16 mM, VC killed approximately 20% and 50% (IC50) of the cancer cells, respectively. Decreased cell viability, increased ROS formation, elevated levels of pro-apoptotic caspases -7, -8, and -9, the decreased expression of cell cycle regulatory proteins CDK2, cyclin B1, and cyclin D1, the reduced expression of MMP2 and MMP9, the increased expression of tumor suppressor genes such as p21, p53, and PTEN, and the decreased expressions of MDM2, p13k, Akt, and mTOR were observed to confirm the effect of a pharmacological dose of VC on TNBC, as presented in Figure 12.

To determine if a pharmacological dose of VC affects another breast cancer cell line, MCF7, an estrogen and progesterone receptor-positive with HER2/neu-negative, was tested, along with non-cancerous cell lines such as kidney HEK 293 and lung CCL205.

The increased nuclear condensation of apoptotic cells was confirmed with Hoechst 33342 staining, showing fewer apoptotic cells at 8 mM compared to the higher 16 mM dose. Additionally, nuclear-ID red/green cell staining reaffirmed that VC at 8 mM and 16 mM induced cell death in MDA-MB-231. MCF7 showed also a similar trend to MDA-MB-231, while non-cancerous HEK293 and CCL205 showed no change with VC treatment of 5 mM or 15 mM.

Evidence suggests that cancer cells generate more intracellular ROS, leading to higher apoptosis rates compared to normal cells [16]. This study demonstrated that VC increased ROS levels, indicating the crucial role of ROS in TNBC cell apoptosis, as confirmed by H2DCF staining. The increased ROS in cancer cells is associated with the modulation of key signaling proteins involved in cell cycle regulation, differentiation, tumor suppression, apoptosis, DNA damage, and the balance between pro-apoptotic and anti-apoptotic genes [17,18]. The upregulation of the pro-apoptotic Bax protein and the downregulation of the anti-apoptotic Bcl2 protein suggest that VC treatment initiates favorable signaling pathways, leading to apoptosis in MDA-MB-231 and MCF7 cells, while it has no effect on HEK293 or CCL205 cells.

The depletion of mitochondrial membrane potential was indicated by Rhodamine 123 staining, causing the increased expression of Apaf-1. Apoptosis in mitochondria also involves several key proteins, such as caspases -7, -8, and -9, highlighting their critical role in cancer cell apoptosis. The collapse of mitochondrial membrane potential results in the release of cytochrome C and other apoptosis-inducing factors into the cytosol, further inducing apoptosis [19,20]. The observed increase in caspases-7, -8, and -9, along with Apaf-1 and cytochrome c, suggests the activation of both intrinsic and extrinsic apoptotic pathways. Cytochrome c release and Apaf-1 upregulation trigger apoptosome formation, leading to caspase-9 activation, while caspase-8, typically linked to death receptor signaling, amplifies the intrinsic pathway by cleaving Bid. Caspase-7, as a downstream effector, executes apoptosis by degrading essential cellular components, demonstrating crosstalk between the pathways [19,20].

Preventing angiogenesis is vital to hindering cancer invasion into surrounding tissues. Matrix-degrading enzymes like matrix metalloproteinases (MMPs)-2 and -9 play crucial roles in cancer invasion, metastasis, and tumorigenesis [21]. A significant decrease in MMP-2 and -9 expression suggests that VC effectively inhibits invasion and metastasis of TNBC cells. This inhibition likely stems from VC’s ability to modulate signaling pathways such as MAPK and NF-κB, which regulate MMP expression. By suppressing these pathways, VC reduces extracellular matrix degradation, thereby impairing cancer cell motility and invasiveness. Additionally, the antioxidant properties of VC may help to counteract reactive oxygen species (ROS) that contribute to tumor progression, further reinforcing its anti-metastatic effects.

Cancer proliferation is often associated with altered cell cycle regulation; hence, targeting cell cycle proteins offers promising cancer chemotherapy strategies [22]. Distinct cell cycle phases (G0/G1, S, G2, and M) are regulated by specific cyclin-dependent kinases (CDKs) [23]. This study showed that significant downregulation of cyclin B1, cyclin D1, and CDK2 expression strongly suggests that VC effectively inhibits MDA-MB-231 cells at different cell cycle phases. There was a slight increase in CDK7 expression (*p* < 0.01) between the control and 8 mM VC treatment; however, no significant change was observed at the higher VC dose of 16 mM, indicating that CDK7 is not affected by VC.

Additionally, increased p21 expression, a CDK inhibitor, confirmed that VC inhibits cell cycle proteins. p21 mediates p53-induced cell cycle arrest, and its induction by p53 and the inhibition of CDKs are crucial for p21’s tumor-suppressive role [24,25].

Furthermore, the enhancement of p21 expression by VC indicates a potential p53-independent mechanism, as p21 can also be regulated by other tumor suppressors and stress-related signaling pathways. The combination of VC-induced downregulation of cyclins and CDKs, along with the upregulation of p21, underscores VC’s capacity to induce cell cycle arrest and provide a strong anti-proliferative effect. These findings highlight the therapeutic potential of vitamin C as an adjunct to traditional chemotherapeutic agents, particularly for targeting the dysregulated cell cycle in aggressive cancers like triple-negative breast cancer [24,25].

In this study, VC was shown to decrease the expression of the p13k/Akt/mTOR pathway, which is often associated with the resistance of cancer cells to treatment using endocrine, HER2-directed, or cytotoxic therapies [26]. Moreover, VC increased pTEN expression, which counteracts p13k activity. The loss of pTEN and mutations in p13k are among the most common aberrations seen in cancers, including breast cancer [26,27]. These alterations contribute to the activation of pro-survival signals, promoting tumor growth and treatment resistance. By re-establishing PTEN expression and inhibiting the pI3K/Akt/mTOR pathway, VC may sensitize breast cancer cells to conventional therapies, potentially overcoming resistance mechanisms. Furthermore, this modulation of the PI3K pathway highlights VC’s role as a promising adjunct in cancer treatment, as it could enhance the efficacy of targeted therapies and reduce the need for higher doses of cytotoxic agents, thereby minimizing adverse effects. The combination of VC with other targeted therapies could offer a strategic approach to overcoming therapeutic resistance and improving patient outcomes in cancers characterized by pI3K pathway dysregulation.

The results of this study suggest that high-dose pharmacological VC induces cell death and inhibits the proliferation of cancerous cells by increasing intracellular ROS, modulating mitochondrial signaling pathways, inhibiting cell cycle progression proteins, preventing angiogenesis, and inhibiting the p13k/Akt/mTOR pathway. The higher dose of VC (16 mM) had a more profound effect than the lower dose (8 mM). This study may have significant implications for the potential application of pharmacological dose of VC in treating TNBC, achieving higher efficacy and displaying potency to kill the cancerous cells while having minimal side effects on normal cells. High-dose VC exerts significant effects on cell cycle proteins, caspase-related proteins, the inhibition of angiogenesis, and the induction of apoptosis.

## 5. Conclusions and Future Directions

This study provides the first evidence that pharmacological concentrations of VC induce the expression of pro-apoptotic proteins such as Apaf-1 and caspases -7 and -9, while inhibiting cancer cell survival proteins like Bcl-2, cyclins D and B1, CDK2, Akt, pI3K, and mTOR. Additionally, VC increases the expression of tumor suppressor genes, including p53, p21, and PTEN. These results offer compelling support for the further exploration of VC as a therapeutic agent for TNBC.

The study also demonstrated that high-dose VC effectively reduces cell viability, inhibits invasion and migration, induces apoptosis, and disrupts mitochondrial membrane potential in MCF-7 breast cancer cells, as well as in MDA-MB-231 cells. Importantly, VC displayed selective cytotoxicity, showing no harmful effects on non-cancerous cells such as HEK 293 and CCL205, highlighting its potential as a safe and effective anti-cancer agent.

Several limitations of this study must be acknowledged. Since the experiments were conducted in vitro using isolated cancer and normal cell lines, the results may not fully capture the complexity of interactions within a living organism, such as the tumor microenvironment, immune responses, and tissue-specific factors. Furthermore, the absence of in vivo validation limits the ability to predict pharmacokinetics, bioavailability, and systemic effects of high-dose VC. Testing was also limited to specific breast cancer subtypes and non-cancerous cell lines, making it unclear whether the observed effects are consistent across other cancer types or normal tissues. Additionally, while the study highlights VC’s therapeutic potential, achieving pharmacological concentrations in clinical settings will likely require intravenous administration, which poses logistical and compliance challenges.

Future research should prioritize addressing these limitations to enhance the clinical relevance of the findings. In vivo studies using animal models are essential to evaluate the therapeutic efficacy and safety of VC under physiological conditions and to explore potential long-term effects. Investigations into the molecular mechanisms underlying VC’s interaction with apoptotic and survival pathways, such as pI3K/Akt/mTOR signaling, will provide deeper insights into its mode of action. Moreover, exploring the synergistic potential of VC in combination with standard chemotherapeutic agents or targeted therapies could pave the way for more effective treatment regimens. Efforts should also focus on understanding whether prolonged exposure to VC may induce resistance mechanisms in cancer cells, helping to optimize treatment protocols. Finally, translating these findings into clinical settings will require well-designed trials to assess dosing regimens, safety, and efficacy in patients with TNBC.

In conclusion, this study lays the groundwork for understanding the anti-cancer potential of pharmacological VC. By addressing these limitations through comprehensive preclinical and clinical studies, VC could emerge as a transformative therapy in the fight against aggressive cancers like TNBC.

## Data Availability

The data are available from the corresponding author upon reasonable request.

## Supplementary Materials

The following supporting information can be downloaded at: https://www.mdpi.com/article/10.3390/biomedicines13030640/s1.

## References

[1] https://www.americanoncology.com/blogs/triple-negative-breast-cancer-symptoms-and-treatment.

[2] Arnold M., Morgan E., Rumgay H., Mafra A., Singh D., Laversanne M., Vignat J., Gralow J.T., Cardoso F., Siesling S. (2022). Current and future burden of breast cancer: Globalstatistics for 2020 and 2040. Breast.

[3] Spano D., Heck C., De Antonellis P., Christofori G., Zollo M. (2012). Molecular Networks that Regulate Cancer Metastasis. Semin. Cancer Biol..

[4] Theossiou T.A., Ali M., Grigalavicius M., Grallert B., Dillard P., Schink K.O., Olsen C.E., Walchli S., Inderberg E.M., Kubin A. (2019). Simultaneous defeat of MCF7 and MDA-MB-231 resistance by a hypericin PDT-tamoxifen hybrid therapy. NPJ Breast Cancer.

[5] Padayatty S.J., Levine M. (2016). Vitamin C Physiology: The Known and the Unknown and Goldilocks. Oral Dis..

[6] Cameron E., Pauling L. (1976). Supplemental Ascorbate in the Supportive Treatment of Cancer: Prolongation of Survival Times in Terminal Human Cancer. Proc. Natl. Acad. Sci. USA.

[7] Shenoy N., Creagan E., Witzig T. (2018). Ascorbic Acid in Cancer Treatment: Let the Phoenix Fly. Cancer Cell.

[8] Cantley L., Yun J. Intravenous High-Dose Vitamin C in Cancer Therapy. https://www.cancer.gov/research/key-initiatives/ras/ras-central/blog/2020/Yun-Cantley-vitamin-C.

[9] Choi Y.K., Kang J., Han S., Kim Y.R., Jo J., Kang Y.W., Choo D.R., Hyun J.W., Koh Y.S., Kang H.K. (2020). L-ascorbic acid inhibits breast cancer growth by inducing IRE/JNK/CHOP-related endoplasmic reticulum stress-mediated p62/SQSTM1 accumulation in the nucleus. Nutrients.

[10] Gan L., Camarena V., Mustafi S., Wang G. (2019). Vitamin C inhibits triple-negative breast cancer metastasis by affecting the expression of YAP1 and synaptopodin 2. Nutrients.

[11] Mussa A., Idris R.A.M., Ahmed N., Ahmad S., Yean C.Y., Rahman W.F.W.A., Lazim N.M., Hajissa K., Mokhtar N.F., Mohamud R. (2022). High-dose vitamin C for cancer therapy. Pharmaceuticals.

[12] Soule H.D., Vazquez J., Long A., Albert S., Brennan M. (1973). A human cell line from a pleural effusion derived from a breast carcinoma. J. Natl. Cancer Institute.

[13] Lacroix M., Leclercq G. (2004). Relevance of breast cancer cell lines as models for breast tumors: An update. Breast Cancer Res. Treat..

[14] Mondal A., Bennett L.L. (2016). Resveratrol enhances the efficacy of sorafenib mediated apoptosis in human breast cancer MCF7 cells through ROS, cell cycle inhibition, caspase 3 and PARP cleavage. Biomed Pharmacother..

[15] Bennett L.L., Mondal A. (2021). Curcumin and afatinib synergisticaly inhibit growth of human osteosarcoma cells by inhibition of matrix metallo proteinases, mitogen activated kinases 1-4, and reactive oxygen species. J. Pharm. Drug Dev..

[16] Simon H.U., Haj-Yehia A., Levi-Schaffer F. (2000). Role of reactive oxygen species in apoptosis induction. Apoptosis.

[17] Mates J.M., Sanchez-Jinenez F.M. (2000). Role of reactive oxygen species in apoptosis: Implications for cancer therapy. Int. J. Biochem. Cell Biol..

[18] Zhang T., Brazhnik P., Tyson J.J. (2007). Exploring the mechanisms of the DNA-damage response: p53 pulses and their possible relevance to apoptosis. Cell Cycle.

[19] Kamalabadi-Farahani M., Najafabadi M.R.H., Jabbarpour Z. (2019). Apoptotic resistance of metastatic tumor cells in triple negative breast cancer: Roles of death receptor-5. Asian Pac. J. Cancer Prev..

[20] Yang Y., Liu X., Bhalla K. (1997). Prevention of apoptosis by Bcl-2 release cytochrome C from mitochondria blocked. Science.

[21] Wang R.X., Chen S., Huang L., Shao Z.M. (2018). Predictive and prognostic value of matrix metalloproteinase (MMP)-9 in neoadjuvant chemotherapy for triple negative breast cancer patients. BMC Cancer.

[22] Otto T., Sicinski P. (2017). Cell cycle proteins as promising targets in cancer therapy. Nat. Rev. Cancer.

[23] Engeland K. (2022). Cell cycle regulation: p53-p21-RB signaling. Cell Death Differ..

[24] Warfel N.A., El-Deiry W.S. (2013). p21WAF1 and tumorigenesis: 20 years after. Curr. Opin. Oncol..

[25] Paplomata E., O’Regan R. (2014). The p13k/AKT/mTOR pathway in breast cancer: Targets, trials and biomarkers. Ther. Adv. Med. Oncol..

[26] Cancer Genome Atlas Network (2012). Comprehensive molecular portraits of human breast tumors. Nature.

[27] Maehama T., Dixon J. (1998). The tumor suppressor, PTEN/MMAC1, dephosphorylates the lipid second messenger, phosphatidylinositol 3,4,5-trisphosphate. J. Biol. Chem..

