# Supplementary material for: Effects of Pharmacological Dose of Vitamin C on MDA-MB-231 Cells
Source: Biomedicines. 2025 Mar 5;13(3):640. doi: 10.3390/biomedicines13030640 (PMC11940700; doi:10.3390/biomedicines13030640)
Supplement: Supplementary file 1 [file biomedicines-13-00640-s001.zip › biomedicines-3486568-supplementary.pdf]

Supplemental MCF western blot effect

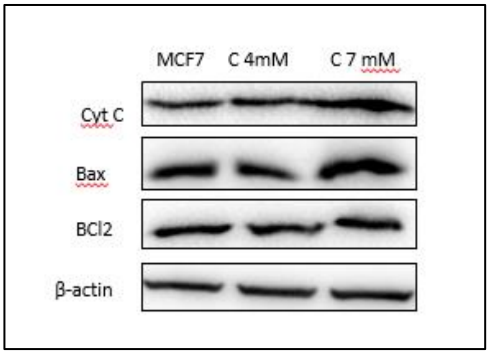

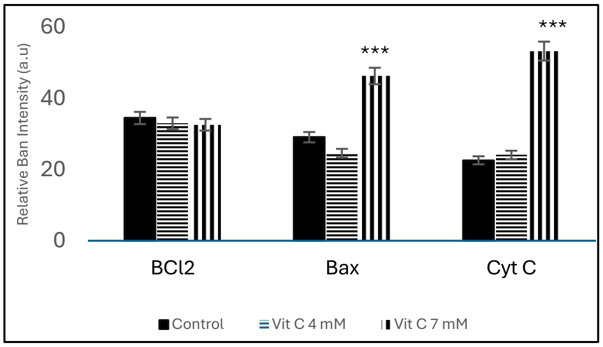

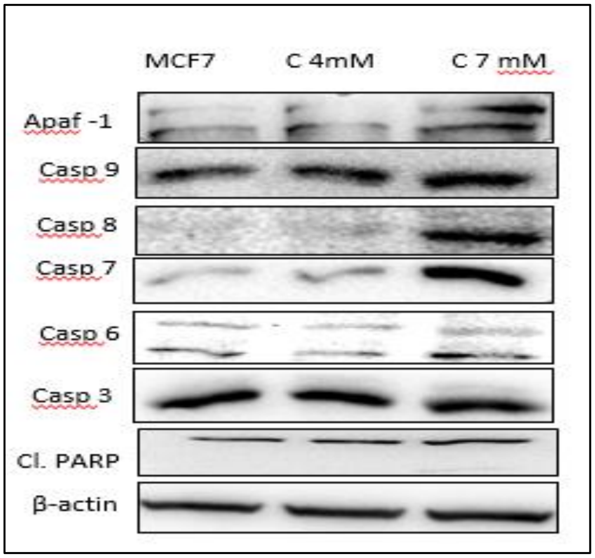

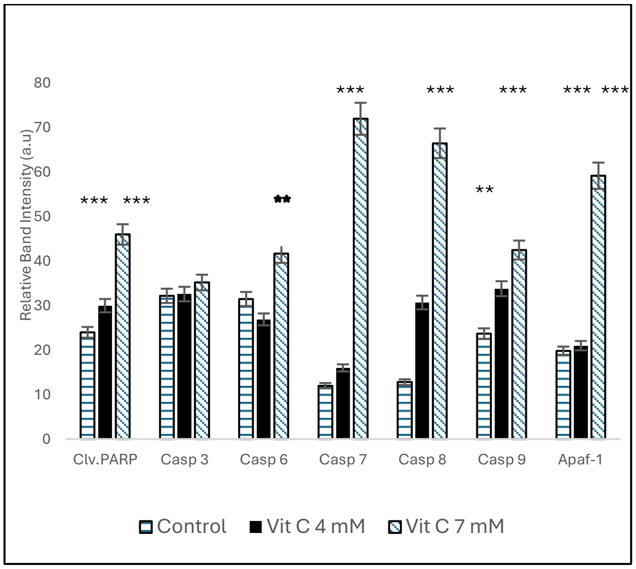

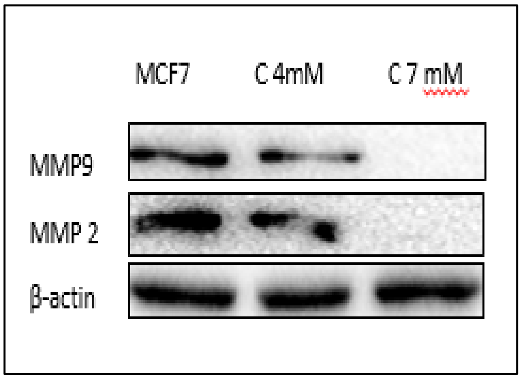

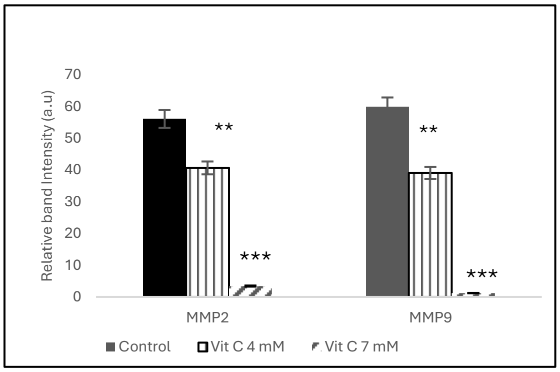

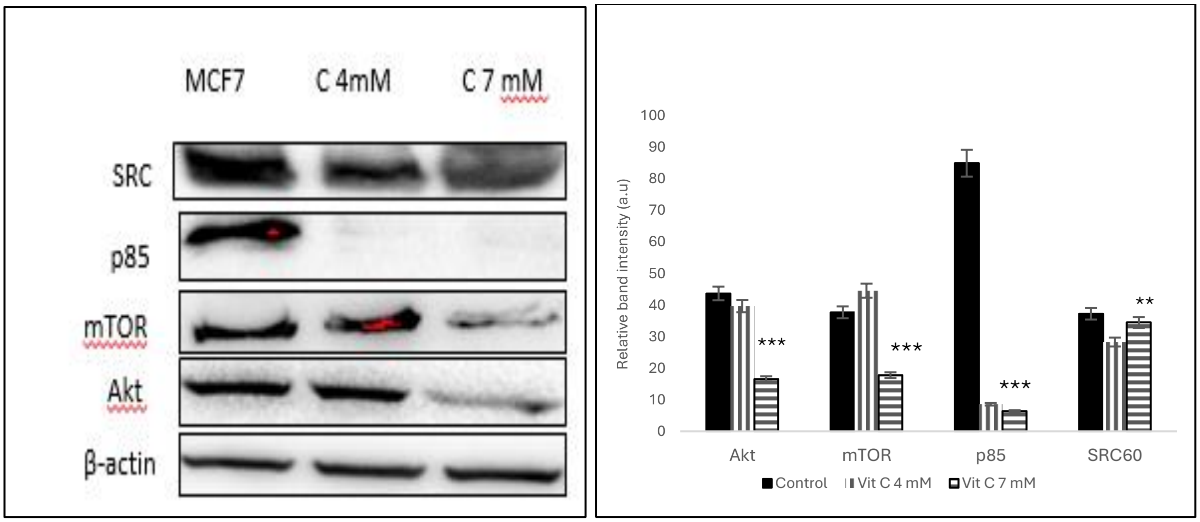

** $p < 0.01$ and ***$p < 0.001$ post hoc- Newman-Keuls test compared to the corresponding control.
